# Improving the dietary quality of food parcels leads to improved dietary intake in Dutch food bank recipients—effects of a randomized controlled trial

**DOI:** 10.1007/s00394-020-02182-8

**Published:** 2020-01-29

**Authors:** Judith E. Neter, S. Coosje Dijkstra, Jos W. R. Twisk, Marjolein Visser, Ingeborg A. Brouwer

**Affiliations:** 1 Department of Health Sciences, Faculty of Science, Vrije Universiteit Amsterdam, Amsterdam Public Health Research Institute, De Boelelaan 1085, 1081 HV Amsterdam, The Netherlands; 2 Department of Epidemiology and Biostatistics, Amsterdam UMC, Location VU University Medical Center, Amsterdam Public Health Research Institute, Amsterdam, The Netherlands

**Keywords:** Nutrition, Dietary intake, Food assistance programs, Low-socioeconomic status, Public health, Intervention

## Abstract

**Purpose:**

Since food banks have a strong influence on recipients’ diets, and seem to have difficulties in supporting healthy diets, improving the dietary quality of food parcels is important. The aim of our study was to assess whether improving the dietary quality of food parcels, using different strategies, can positively impact the actual dietary intake of Dutch food bank recipients.

**Methods:**

This randomized cross-over controlled trial (Trial ID: ISRCTN40554133) with four intervention conditions [(1) Control (standard food parcel), (2) snacks^–^ (standard food parcel with replacement of unhealthy snacks by staple foods), (3) FV^+^ (standard food parcel plus the recommended daily amount of fruit and vegetables), (4) snacks^–^ + FV^+^ (standard food parcel with replacement of unhealthy snacks by staple foods plus the recommended daily amount of fruit and vegetables)] included 163 food bank recipients, from three food banks. At baseline, participants filled in a questionnaire. Dietary intake data were collected through 24-h recalls after both intervention conditions at 4 and 8 weeks follow-up. Primary outcome was daily fruit and vegetable intake, secondary outcomes were daily dietary intakes of food groups and nutrients.

**Results:**

Multi-level linear regression analysis, using a two-level model, showed a higher mean daily fruit intake in participants in the FV^+^ condition than in participants in the Control condition (delta (δ): 74 [40.3;107.6] g). Both mean daily fruit and vegetable intake were higher in participants in the Snacks^–^ + FV^+^ condition than in participants in the Control condition (fruit δ: 81.3 [56.5;106.2] g; vegetables δ: 46.2 [17.5;74.9] g), as well as in the Snacks^–^ condition (fruit δ: 70.0 [38.8;101.1] g; vegetables δ: 62.2 [26.2; 98.2] g).

**Conclusions:**

This study shows that improving the dietary quality of food parcels can positively impact the dietary intake of Dutch food bank recipients. With this information we can further develop effective strategies that can be easily applied by food banks, to improve dietary intake of food bank recipients.

## Introduction

Food assistance program users are generally low-income families and individuals, who make use of temporary food assistance programs such as food banks, food pantries, or a supplemental nutrition assistance program. Food assistance program users often have poorer diet quality compared to people not using any food assistance programs (non-users) [1–3]. Studies on dietary intake of food assistance program users show that they have a lower consumption of fruit, vegetables, dairy products, and seafood than recommended by the national dietary guidelines [2, 4], and also when compared to the general population [5] and to the low-socioeconomic status (SES) population [2, 6].

Dutch food banks aim to weekly provide food parcels that supplement the normal diet for 2–3 days. The content of these food parcels largely depends on donated foods by food companies, supermarkets and individuals. As food bank recipients have limited resources to purchase additional foods, they largely rely on the quantity and quality of the food in the food parcels [7–10]. Previous studies have shown that the content of food parcels is often not in line with dietary guidelines [11, 12]. Our previous work also shows that the content of food parcels supplied by the Dutch food bank provided too high amounts of energy, protein and saturated fat, whereas the provided amounts of fruits and fish were too low to meet the Dutch dietary guidelines [13]. These results suggest that food bank recipients cannot meet the dietary guidelines for a healthy diet if the food provided in the food parcels is the major food source. This consequently may lead to higher risks of nutrition-related chronic diseases. Food bank recipients are thus a specific group of nutritional concern.

Since food banks have a strong influence on recipients’ diets, and nowadays seem to have difficulties in supporting healthy diets, improving the dietary quality of the food parcels is important. A modelling study, using linear programming, showed that dietary recommendations for macro- and micronutrients could be better met by improving the content of the food parcel [14]. However, it is unclear whether improving the dietary quality of the food parcels will actually lead to a healthier dietary intake among their users, and it is unknown what the best strategy is to improve the parcels. Therefore, we aim to assess whether improving the dietary quality of the food parcels, using different strategies, will positively impact the actual dietary intake of Dutch food bank recipients. This knowledge is necessary to help develop the most effective strategy that can be applied by food banks to improve dietary intake of Dutch food bank recipients.

## Methods

At the start of the study in 2012, there were approximately 135 food banks in the Netherlands. The inclusion criterion for food banks to participate in our study was provision of food parcels once a week. To increase the representativeness, we recruited three large food banks (> 100 recipients) in total, from medium size (< 150,000 inhabitants) as well as larger cities (≥ 150,000 inhabitants), and from different regions across the Netherlands (provinces: Noord-Holland, Gelderland and Overijssel), which were willing to participate.

This study was approved by the Medical Ethical Committee of the VU Medical Center in Amsterdam, the Netherlands, as well as the national board of the Dutch Food Bank. The study is registered at the ISRCTN trial register (ISRCTN40554133). The CONSORT checklist was used to avoid inadequate reporting of the study.

### Design

**Fig. 1:**
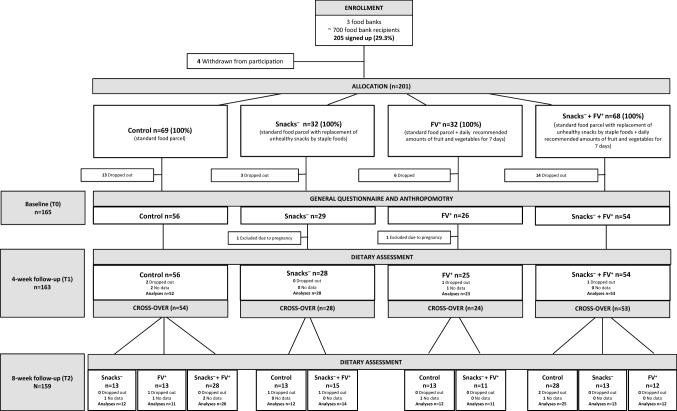
Flowchart of recruitment, allocation, measurements and follow-up of the study participants

We performed a randomized cross-over controlled trial because fewer participants are needed compared to a parallel design. Food bank recipients are participants that have a lot going on in their lives and therefore are hard to recruit. Furthermore, this design makes it possible to study different strategies within the same participants. The trial included 4 intervention conditions in two consecutive periods, lasting 4 weeks each. We did not include a wash-out period because we expected little to no carry-over effect; participants received food parcels weekly and dietary intake data was collected within a week after receiving the food parcel. There is a chance that participants kept the non-perishable foods, but since we previously showed that low food security is highly prevalent [15] we assume this is a very small chance. Per food bank, the trial lasted 8 weeks in total. Food parcels were supplied during 10 weeks, but final measurements took place 2 weeks before the end of the intervention. Data were collected between October 18th and December 20th, 2012. Per food bank, participants were randomly allocated to one of the possible sequences by the researchers, before the start of the trial. Not all intervention conditions were comparable because it would not be informative to compare two completely different interventions. At baseline (T0, after randomization), participants filled in a questionnaire and underwent anthropometric measurements. Dietary intake data were collected through 24-h (24-h) recalls after both intervention conditions at 4 (T1) and 8 (T2) weeks follow-up. A flowchart of the study is presented in Fig. 1.

### Recruitment and participant selection

Recipients of the three food banks were recruited through information letters, active recruitment at the food banks and promotional posters between October 5th and 18th, 2012. The aim of the study on the recruitment materials was to assess improvement of food parcels. The recipients could sign up for the study with an application form, by telephone or e-mail. Of the approximately 700 food bank recipients at the three participating food banks, 205 (29%) voluntarily indicated that they were interested to participate (Fig. 1). Food bank recipients were eligible to participate if they met the following criteria: (1) ≥ 18 years of age, (2) adequate command of the Dutch language to participate in oral and written interviews, (3) recipient of a Dutch food bank > 1 month, (4) collect own food parcel at the food bank, and (5) possible to be contacted by phone. Only one member per household was enrolled. Four recipients withdrawn from participation. In total, 201 food bank recipients were randomly allocated to one of the ten possible sequences. Forty recipients signed up for participation, but ultimately did not participate. For 22 of these recipients we were able to ascertain the reason for non-participation; (1) no longer a food bank recipient at the start of the study (*n* = 5), (2) lack of time (*n* = 5), (3) no longer wanted to participate (*n* = 2), (4) no adequate command of the Dutch language (*n* = 4), and (5) other reasons (*n* = 6) such as illness or not willing to participate in anthropometric measurements. Participants who completed the study received a gift coupon of 5 Euros and a bag with fruit and vegetables and non-food items (e.g., personal care products and tools to make it easier and more fun to prepare the fruits and vegetables).

### Development intervention

Development of the intervention was based on the results of our previous studies in which we showed that (1) the provided amounts of saturated fat in a single-person food parcel for one single day were higher, whereas the provided amounts of fruits were lower than the nutritional guidelines [13], and (2) that the majority of the Dutch food bank recipients had lower intakes than dietary reference intakes for dietary fiber, fruit, vegetables, and fish, and a higher intake for saturated fat [16]. Based on these results, we expected to be able to improve dietary intake by providing extra fruit and vegetables to the food parcel, and remove unhealthy snacks from the food parcel. Potential intervention strategies to improve the content of the food parcels were also discussed with participants during focus group discussions. Strategies that participants seemed to be most enthusiastic about and were considered feasible and effective in improving dietary intake by the researchers were selected as intervention conditions for this study. Prior to the main study, a pilot was carried out among 19 food bank recipients from a food bank in a small city (Huizen, the Netherlands) to test our study materials and the feasibility of the intervention strategies. This was done by supplying altered food parcels, administering a questionnaire, measuring dietary intake through 24-h recalls and obtaining anthropometric measurements.

### Intervention conditions

The trial consisted of the following intervention conditions:

(1) Control: the standard food bank specific food parcel with additional non-food items (e.g., personal care products, blanket).

(2) Snacks^–^: the standard food bank specific food parcel in which unhealthy snacks (e.g., chocolate, cookies, potato chips) were replaced by staple foods (e.g., pasta, rice), with additional non-food items (e.g., personal care products, blanket).

(3) FV^+^: the standard food bank specific food parcel plus the recommended daily amount of fruit (2 pieces) and vegetables (200 g) [17] for all household members for 7 days (4 days fresh, and 3 days non-perishable vegetables).

(4) Snacks^–^ + FV^+^: the standard food bank specific food parcel in which unhealthy snacks (e.g., chocolate, cookies, potato chips) were replaced by staple foods (e.g. pasta, rice) plus the recommended daily amount of fruit (2 pieces) and vegetables (200 g) [17] for all household members for 7 days (4 days fresh, and 3 days non-perishable vegetables).

The total amount of kilocalories of the food parcels was not taken into account in improving the dietary content of the food parcels. Essential non-food items were added to the food parcels in the Control and Snacks^–^ conditions to let participants in these conditions also benefit from their participation in the intervention study.

### Measurements

#### Questionnaire

At baseline, participants completed a self-administered general questionnaire at the food bank, which consisted of questions regarding socio-demographics and smoking. Participants who had difficulties in reading or writing were offered help filling in the questionnaire.

Socio-demographics included date of birth, sex, duration of being recipient of the Dutch food bank (< 6 months, 6–12 months, and > 12 months), household composition (number of children < 18 years, ≥ 18 years, and adults in household) and highest completed educational level. For household composition, we created three categories: single parent household (including one adult and at least one child), single or multiple household without children (including at least one adult and no children), and multiple household with children (including at least two adults and at least one child). We created three levels of education: low (did not complete any education or finished elementary school), medium (high school, general intermediate, and lower vocational education, general secondary, and intermediate vocational education) and high (higher vocational education, university). Smoking was categorized into current smoker yes or no.

#### Anthropometric measurements

At baseline, trained researchers measured participants body height and body weight, according to a standardized protocol developed for this study. For these measurements, participants were asked to remove any items from their pockets and to take off their shoes and coat. Not all participants (*n* = 4) were willing to take off their shoes and / or coat. For these persons we adjusted height and weight using standardized measures for clothes and shoes, based on the study of Frank et al. [18]. A portable stadiometer, type Seca ® 214, was used to measure height to the nearest 0.1 cm, and a calibrated mechanical scale, type Seca ® 761, to measure weight to the nearest 0.5 kg. Body mass index (BMI) was calculated as measured weight (kg) divided by measured height squared (m^2^). BMI cutoff points of the WHO were used to define weight status [19].

#### Study outcome: dietary intake

Dietary intake data were collected by trained interviewers through multiple 24-h recalls, after both intervention conditions at 4 (T1) and 8 (T2) weeks follow-up, using the USDA five-step Multiple-Pass Method (MPM) [20–22]. The MPM method was developed for collecting interviewer-administered 24-h recalls and includes multiple passes through the 24-h of the previous day, during which respondents receive cues to help them remember and describe foods and drinks they consumed [22]. Interviewers additionally asked participants whether the recall day was a normal day regarding dietary intake (yes/no).

During the baseline measurements of the study at T0 a table scale, type KERN FCE 6 K2 ® and extensive tableware were used, and a portion size photo booklet was introduced to the participants to assist in portion-size estimation of consumed foods and drinks at T1 and T2. The portion size photo booklet was taken home by the participants to be used in the 24-h recalls, which were all conducted by phone at dates and times unannounced to the participants. We aimed to obtain dietary information on two different weekdays and one weekend day, or if not possible, on three different weekdays.

All recorded foods and drinks from the multiple 24-h recalls per intervention period were coded with the corresponding Dutch Food Composition Table code (NEVO-code) [23, 24]. Portion sizes consumed were entered in grams [23]. Energy content (kcal) and nutrient composition (mg, g, en%) of the foods and drinks consumed were determined using the 2010 NEVO-database [24], which contains the nutrient composition of foods and drinks commonly consumed in the Netherlands. The primary outcomes of the intervention were mean daily fruit and vegetable intake (g/d). Secondary outcomes were mean daily dietary intake in terms of food groups [grains, flour, rice (g/d), nuts, seeds and snacks (g/d), pastry and cookies (g/d), pulses (g/d), and sugar, candy, sweet filling and sweet sauces (g/d)] and nutrients [energy (kcal/d), protein (en%/d), mono- and disaccharides (en%/d), polysaccharides (en%/d), total fat (en%/d), saturated fat (en%/d), fiber (g/d), vitamin C (mg/d), sodium (g/d), and potassium (mg/d)]. Amount of foods from food groups consumed (g), and nutrient data (mg, g) were averaged over the days assessed.

### Blinding

Due to the nature of the intervention, it was not possible to blind participants to the allocated intervention conditions. Researchers were not blinded to the allocated intervention conditions of the participants because they weekly prepared the content of the food parcels and supplied the food parcels themselves. Therefore, there was intensive personal contact with the participants, which made blinding not possible.

### Power calculation

The sample size was calculated using data on fruit and vegetable intakes from the Dutch National Food Consumption Survey [25]. To detect a difference of 60 grams of fruit between two conditions (assuming a standard deviation of 114 grams per day [25]), a power of 80%, a significance level of 5% and a correlation between the repeated measurements of 0.6), 40 subjects per condition were needed. With 40 subjects for each condition, a difference of 52 grams per day in vegetable intake (assuming a standard deviation of 95 grams per day [25]) can be detected.

### Statistical analyses

Statistical analyses were performed using IBM SPSS statistics for Windows version 21.0 (Armonk, NY: IBM Corp, USA) and STATA (version 14). Descriptive statistics were used to summarize participants’ characteristics. Continuous variables were presented as mean and standard deviation (SD), whereas categorical variables were presented as frequency and relative frequency.

Differences in dietary intake between the four intervention conditions were assessed using multi-level linear regression analysis, using a two-level model (i.e., repeated measurements clustered within participants). In the first model, crude analyses were conducted, while in the second model adjustments were made for order of the intervention (period 1 or period 2), the number of days between receiving the last food parcel and the 24-h recall, food bank, and whether the recall day was a normal day regarding dietary intake (yes/no). In addition, we tested for interaction between the intervention conditions and baseline body weight status of the participants (underweight/normal weight vs. overweight/obesity). Differences in mean daily intake between the intervention conditions are presented in kilocalories, (milli)grams, or energy percentages and their corresponding 95% confidence intervals (95%CI). All analyses were intention-to-treat.

## Results

### Participant characteristics

Of the food bank recipients who signed up for the study (*N* = 205), 165 (80.5%), completed the baseline measurements (Fig. 1). Two females who reported being pregnant at baseline were excluded from data analysis. Consequently, a total of 163 participants were included for data analyses. Nine participants dropped out during the intervention study; eight because they were no longer recipient of the food bank and one because of personal reasons.

The three participating food banks (with number of participants indicated in brackets) were located in Alkmaar (*n *= 33), Apeldoorn (*n* = 43), and Enschede (*n *= 87). Mean age of the study population was 45.1 (SD:10.8) years and 68.1% was female (Table 1). The majority of the participants lived in households without children (52.5%), and was educated at medium level (73.6%). Furthermore, 65% was a current smoker and almost 60% was either overweight or obese.

**Table 1:** Baseline characteristics of the 163 Dutch food bank recipients participating in the intervention study^a^

Characteristics	
Age, years	45.1 ± 10.8
Sex	
Male	52 (31.9)
Female	111 (68.1)
Duration of being recipient	
< 6 months	56 (34.4)
6–12 months	52 (31.9)
≥ 12 months	55 (33.7)
Household composition*	
Single parent household	46 (28.4)
Single or multiple household without children	85 (52.5)
Multiple household with children	31 (19.1)
Educational level	
Low	28 (17.2)
Medium	120 (73.6)
High	15 (9.2)
Current smoking	
Yes	106 (65.0)
No	57 (35.0)
BMI^b*^, *kg/m*^*2*^	28.2 ± 6.8
Weight status^b^*	
Underweight; BMI < 18.5 kg*/*m^2^	7 (4.3)
Normal weight; BMI 18.5–24.9 kg/m^2^	59 (36.4)
Overweight; BMI 25–29.9 kg/m^2^	43 (26.5)
Obese; BMI ≥ 30 kg*/*m^2^	53 (32.7)

^a^Values are presented as mean ± SD, as frequency with between brackets the relative frequency as a percentage

^b^Based on measured height and weight

^*^Household composition, BMI, weight status *N *= 162

### Dietary intake

At T1, we assessed dietary intake of 16 participants using one, 119 participants using two, and 21 participants using three 24-h recalls. At T2, these were 20, 127 and 0 participants, respectively. We were not able to assess dietary intake of 2 participants at T1 and 7 participants at T2. Mean daily dietary intakes of food groups and nutrients, stratified by intervention condition, are presented in Table 2.

**Table 2:** Mean daily dietary intake (SD) of food groups and nutrients, stratified by intervention condition, of 159 Dutch food bank recipients^a,b,^^c^

	Control *N* = 101	Snacks^–^ *N* = 53	FV^+^ *N* = 46	Snacks^–^ + FV^+^ *N* = 104
*Food groups*				
Fruit, *g*	65.2 ± 106.1	69.4 ± 126.7	130.7 ± 152.9	153.8 ± 166.7
Vegetables, *g*	89.9 ± 129.2	64.4 ± 100.6	108.6 ± 129.3	144.3 ± 226.2
Grains, flour, rice, *g*	52.4 ± 115.0	30.0 ± 87.0	62.4 ± 137.1	74.0 ± 289.4
Nuts, seeds and snacks, *g*	19.1 ± 48.9	26.2 ± 56.4	22.7 ± 73.0	24.1 ± 60.1
Pastry and cookies, *g*	21.2 ± 44.4	31.7 ± 77.2	19.6 ± 46.3	27.5 ± 51.4
Pulses, *g*	5.7 ± 30.1	23.7 ± 93.3	14.9 ± 86.9	10.2 ± 47.0
Sugar, candy, sweet filling and sweet sauces, *g*	29.6 ± 43.0	48.9 ± 70.1	32.1 ± 36.8	30.9 ± 41.8
*Nutrients*				
Energy, *kcal*	1872 ± 1038	2212 ± 1368	1947 ± 1035	2154 ± 1738
Protein, *en%*	15.5 ± 4.9	14.1 ± 4.2	14.2 ± 5.2	15.1 ± 5.1
Carbohydrates,* en%*	45.5 ± 13.1	46.1 ± 12.9	51.0 ± 15.9	46.9 ± 11.8
Mono- and disaccharides, *en%*	21.5 ± 13.5	21.6 ± 12.1	27.6 ± 18.5	23.7 ± 11.8
Polysaccharides, *en%*	24.0 ± 8.8	24.4 ± 9.7	23.4 ± 9.3	23.2 ± 7.3
Fat total, *en%*	35.1 ± 11.8	35.5 ± 12.0	30.8 ± 12.2	33.4 ± 10.4
Fat saturated, *en%*	13.0 ± 5.3	13.1 ± 5.2	12.0 ± 5.3	12.5 ± 4.5
Fiber, *g*	14.8 ± 9.9	17.2 ± 13.0	17.0 ± 11.7	18.4 ± 12.6
Vitamin C, *mg*	71.7 ± 99.2	66.4 ± 80.9	83.5 ± 79.3	98.3 ± 128.2
Sodium, *g*	2.2 ± 1.5	2.7 ± 1.8	2.2 ± 1.5	2.4 ± 2.4
Potassium, *mg*	2838 ± 1637	2927 ± 1501	3088 ± 1627	3452 ± 2258

^a^The number of participants per intervention condition is the sum of participants in the conditions in intervention period I and intervention period II. Therefore, it does not add up to the total number of participants (Fig. 1)

^b^Values are presented as mean ± SD

^c^*Control condition* standard food bank specific food parcel with additional non-food items; *Snacks*^*–*^* condition*: standard food bank specific food parcel in which unhealthy snacks were replaced by staple foods, with additional non-food items; *FV*^+^ *condition*: standard food bank specific food parcel with additionally the recommended daily amount of fruit and vegetables for all household members for 7 days; *Snacks*^*–*^ + *FV*^+^ *condition*: standard food bank specific food parcel in which unhealthy snacks were replaced by staple foods with additionally the recommended daily amount of fruit and vegetables for all household members for 7 days

### Differences in mean daily food group intake

**Fig. 2:**
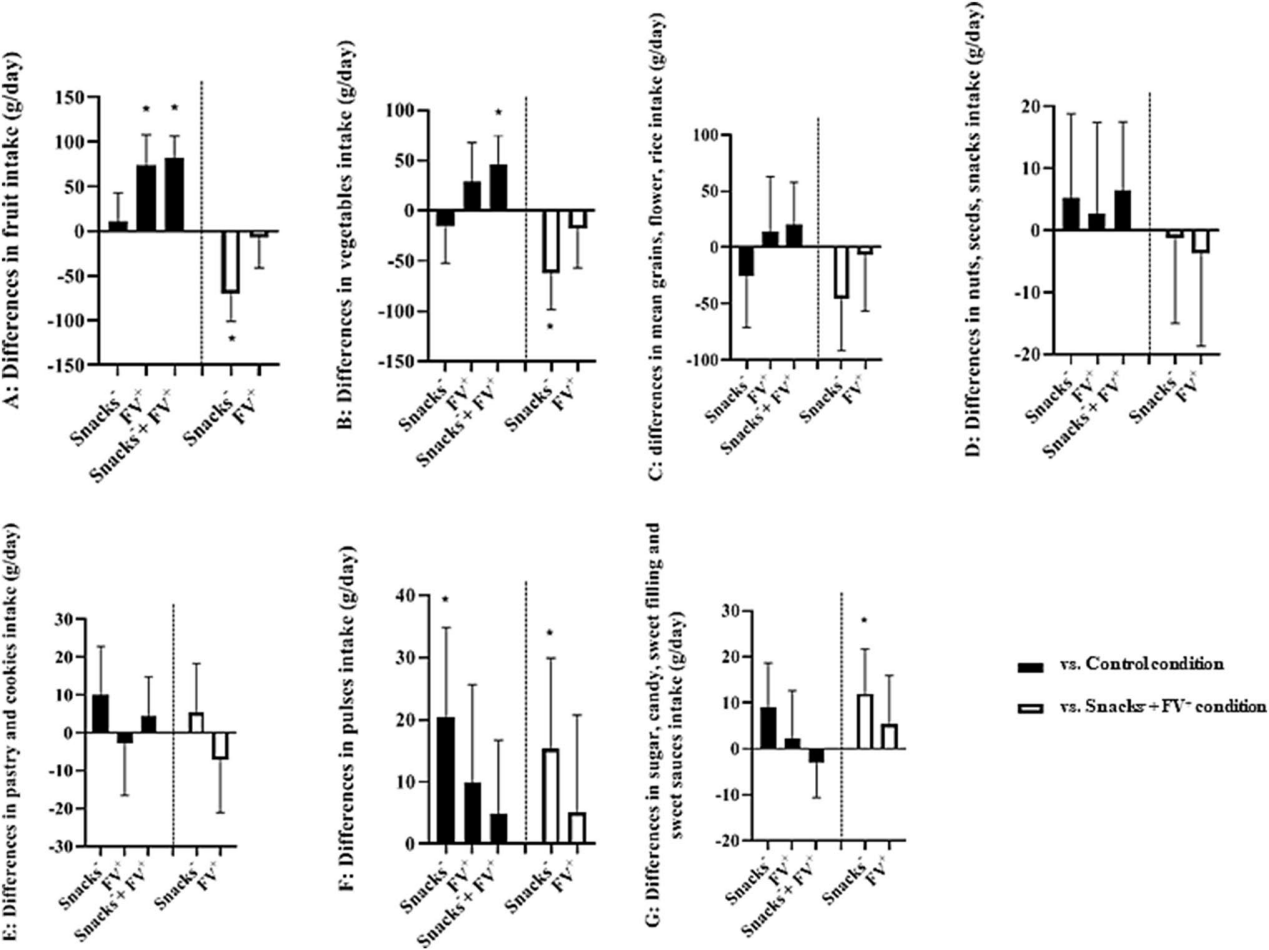
Estimated differences^1^ in mean daily product group (A-G) intake in grams with their corresponding 95% confidence intervals per intervention condition^2^, compared to the reference group. Black bars represent differences in mean daily intake compared to the control condition, white bars represent differences in mean daily intake compared to the Snacks^–^ + FV^+^ condition. ^1^Multilevel linear regression analysis were adjusted for: number of days between receiving the last food parcel and 24-h recall, order of interventions, food bank and whether the day of the 24-h recall was a normal day regarding dietary intake. ^2^*Control condition* standard food bank specific food parcel with additional non-food items; *Snacks*^*–*^* condition* standard food bank specific food parcel in which unhealthy snacks were replaced by staple foods, with additional non-food items; *FV*^+^ *condition*: standard food bank specific food parcel with additionally the recommended daily amount of fruit and vegetables for all household members for 7 days; *Snacks*^*–*^ + *FV*^+^ *condition*: standard food bank specific food parcel in which unhealthy snacks were replaced by staple foods with additionally the recommended daily amount of fruit and vegetables for all household members for 7 days. *P < 0.05

Figure 2 shows adjusted differences in mean daily food group intake (A-G) for each intervention condition versus the reference group (δ). Mean daily intake of pulses (δ: 20.3 [95%CI 5.8;34.8] g) was higher in the Snacks^–^ condition than in the Control condition. In the FV^+^ condition, mean daily fruit intake (δ: 74 [40.3;107.6] g) was higher than in the Control condition. Both mean daily fruit (δ: 81.3 [56.5;106.2] g) and mean daily vegetable intake (δ: 46.2 [17.5;74.9] g) were higher in the Snacks^–^ + FV^+^ condition than in the Control condition.

In the Snacks^–^ condition, mean daily intakes of fruit (δ:  − 70.0 [ − 101.1; − 38.8] g) and vegetables (δ:  − 62.2 [ − 98.2; − 26.2] g) were lower than in the Snacks^–^ + FV^+^ condition, whereas mean daily intakes of pulses (δ: 15.4 [0.9;29.9] g), sugar, candy, sweet filling and sweet sauces (δ: 12.0 [2.4;21.7] g) were higher than in the Snacks^–^ + FV^+^ condition. The latter difference was most attributable to participants with underweight/normal weight compared to participants with overweight/obesity. No statistically significant differences were found in mean daily food group intakes between the FV^+^ condition and the Snacks^–^ + FV^+^ condition.

### Differences in mean daily nutrient intake

Results of the adjusted multilevel analyses show statistically significant differences in mean daily intakes of several nutrients between the different intervention conditions (Table 3). In the Snacks^–^ condition, mean daily intake of protein (difference (δ):  − 1.3 [ − 2.4; − 0.2] en%) was lower than in the Control condition. In the FV^+^ condition, mean daily intakes of carbohydrates (δ: 4.9 [1.8;8.0] en%), mono- and disaccharides (δ: 4.8 [1.8;7.8] en%) and fiber (δ: 2.8 [0.1;5.4] g) were higher, whereas mean daily intake of total fat (δ:  − 4.5 [ − 7.4; − 1.7] en%) was lower than in the Control condition. The differences in the intakes of carbohydrates and total fat were most attributable to participants with overweight/obesity, compared to their counterparts. Similarly statistically significant differences were found in mean daily intakes of carbohydrates, mono- and disaccharides, total fat and fiber between the Snacks^–^ + FV^+^ condition and the Control condition. Additionally, mean daily intakes of vitamin C (δ: 22.2 [2.6;41.8] mg) and potassium (δ: 427 [121;734] mg) were higher in the Snacks^–^ + FV^+^ condition than in the Control condition.

**Table 3:** Estimated adjusted differences in mean daily nutrient intake per intervention condition, compared to the Control and the Snacks^–^ + FV^+^ conditions^a,b,c,d^

	Snacks^–^ (95%CI) *N* = 53	FV^+^ (95%CI) *N* = 46	Snacks^–^ + FV^+^ (95%CI) *N* = 104	Snacks^–^ (95%CI) *N* = 53	FV^+^ (95%CI) *N* = 46
	*vs.* control, *N* = 101			*vs.* Snacks^–^ + FV^+^, *N* = 104	
Energy, *kcal*	− 9 ( − 295;277)	63 ( − 245;372)	146 ( − 79;371)	− 155 ( − 440;129)	− 82.8 ( − 396;231)
Protein, *en%*	− 1.3 ( − 2.4; − 0.2)*	− 0.8 ( − 1.9;0.4)	− 0.5 ( − 1.4;0.3)	− 0.8 ( − 1.9;0.3)	− 0.3 ( − 1.4;0.9)
Carbohydrates, *en%*	2.1 ( − 0.8;5.0)	4.9 (1.8;8.0)*	2.8 (0.6;5.1)*	− 0.7 ( − 3.6;2.2)	2.1 ( − 1.1;5.2)
Mono- and disaccharides, *en%*	1.2 ( − 1.6;4.0)	4.8 (1.8;7.8)*	2.9 (0.7;5.1)*	− 1.7 ( − 4.5;1.1)	2.0 ( − 1.1;5.0)
Polysaccharides, *en%*	1.0 ( − 1.0;3.0)	− 0.1 ( − 2.3;2.0)	− 0.3 ( − 1.9;1.3)	1.3 ( − 0.7;3.3)	0.2 ( − 2.0;2.3)
Fat total, *en%*	− 0.9 ( − 3.6;1.7)	− 4.5 ( − 7.4; − 1.7)*	− 2.4 ( − 4.5; − 0.3)*	1.5 ( − 1.1;4.1)	− 2.1 ( − 5.0;0.8)
Fat saturated, *en%*	− 0.3 ( − 1.4;0.9)	− 1.2 ( − 2.4;0.1)	− 0.5 ( − 1.4;0.4)	0.2 ( − 0.9;1.4)	− 0.7 ( − 1.9;0.6)
Fiber, *g*	1.4 ( − 1.1;3.8)	2.8 (0.1;5.4)*	2.6 (0.7;4.6)*	− 1.3 ( − 3.7;1.2)	0.2 ( − 2.6;2.9)
Vitamin C, *mg*	− 9.4 ( − 33.8;15.0)	17.7 ( − 8.5;44)	22.2 (2.6;41.8)*	− 31.6 ( − 56; − 7.2)*	− 4.5 ( − 31;22.1)
Sodium, *g*	0.1 ( − 0.3;0.5)	0.0 ( − 0.4;0.4)	0.1 ( − 0.2;0.4)	0.0 ( − 0.4;0.4)	− 0.1 ( − 0.5;0.4)
Potassium, *mg*	− 265 ( − 654;124)	397 ( − 22.2;817)	427 (121;734)*	− 692 ( − 1079; − 305)*	− 30.1 ( − 456;396)

^a^The number of participants per intervention condition is the sum of participants in the conditions in intervention period I and intervention period II. Therefore, it does not add up to the total number of participants (Fig. 1)

^b^Values are presented as mean differences and 95%CI

^c^Adjusted for number of days between receiving the last food parcel and 24-h recall, order of interventions, food bank and whether the day of the 24-h recall was a normal day regarding dietary intake

^d^*Control condition* standard food bank specific food parcel with additional non-food items; *Snacks*^*–*^* condition* standard food bank specific food parcel in which unhealthy snacks were replaced by staple foods, with additional non-food items; *FV*^+^ *condition*: standard food bank specific food parcel with additionally the recommended daily amount of fruit and vegetables for all household members for 7 days; *Snacks*^*–*^ + *FV*^+^ *condition*: standard food bank specific food parcel in which unhealthy snacks were replaced by staple foods with additionally the recommended daily amount of fruit and vegetables for all household members for 7 days

^*^Significantly differ from the reference group

Except from lower mean daily intakes of vitamin C (δ:  − 31.65 [ − 56; − 7.2] mg) and potassium (δ:  − 692 [ − 1079; − 305] mg) in the Snacks^–^ condition than in the Snacks^–^ + FV^+^ condition, no statistically significant differences in mean daily intakes between these two intervention conditions were found. No statistically significant differences were found in mean daily nutrient intake between the FV^+^ condition and the Snacks^–^ + FV^+^ condition.

## Discussion

To the best of our knowledge, this study is the first randomized controlled trial to compare multiple strategies improving the dietary quality of food parcels on dietary intake in food bank recipients. Results of this study show that, overall, adding fruit and vegetables to the standard food bank specific food parcels (either in combination with replacing unhealthy snacks from the standard food parcel by staple foods or not) positively impacted dietary intake of Dutch food bank recipients. Participants in the FV^+^ condition showed higher mean daily fruit intake than participants in the Control condition. Both mean daily fruit and mean daily vegetable intake were statistically significant higher in participants in the Snacks^–^ + FV^+^ condition than in participants in the Control as well as in the Snacks^–^ condition. The intervention strategy in which unhealthy snacks from the standard food parcel were replaced by staple foods did not improve dietary intake of the participants; mean daily intake of the food group nuts, seeds and snacks did not significantly differ from the reference groups and mean daily intake of the food group sugar, candy, sweet filling and sweet sauces was statistically significant higher than in the Snacks^–^ + FV^+^ condition.

It is not possible to directly compare our results with other studies conducted in food assistance programs, as our study is the first to examine the effect of changing the actual content of the food parcels in various ways. In contrary to our results, a randomized intervention study by Depa et al. [26], in which two daily portions of FV each week for four weeks were provided, did not show higher FV intakes in food bank users compared to non-users. This difference might be explained by the differences in the amount of fruit and vegetables provided, sample size and method used to assess dietary intake (food frequency questionnaire vs. 24-h dietary recalls). Other studies have mainly focused on improving the diet through financial incentives or restriction of purchase of less nutritious foods. A randomized clinical trial in low income participants of a food benefit program [27] showed that a financial incentive on fruit and vegetables (i.e., 30% of the purchase price) resulted in a higher fruit but not vegetable intake compared to the control group. A randomized controlled trial by Olsho et al. [28] also showed a higher fruit intake, but also a higher vegetable intake in Supplemental Nutrition Assistance Program (SNAP) participants who received a 30% rebate on fruit and vegetable purchased with SNAP benefits. Making fruit and vegetable consumption easier, by either adding fruit and vegetables to the food parcels as we did in our study, or reducing the price of fruit and vegetables in other studies, seems to result in higher fruit intakes, but not always in higher vegetable intakes. Adding fruit and vegetable to the food parcel seems to be the more effective in improving fruit (δ 74–81.3 g/d) and vegetable (δ 46.2–62.2 g/d) intake in food assistance program users than a financial incentive of 30% (fruit: δ 26–40 g/d; vegetables: δ 30.8 g/d) [27, 28].

Harnack et al. [27] also showed that restricting the purchase of less nutritious foods (i.e., sugar sweetened beverages, candies and sweet baked goods) in combination with a financial incentive on fruit and vegetables resulted in lower servings of the restricted foods per day compared to both the control group and the group receiving a financial incentive on fruit and vegetables. On the other hand, restriction of purchase of less nutritious foods only did not always result in lower intakes of less nutritious foods. In our study, we did not find an effect of replacing unhealthy snacks from the standard food parcel by staple foods either in combination with additional fruit and vegetables or not, on snack intake. Although the participants in the study by Harnack et al. [27] were lower income participants who were not currently enrolled in SNAP the study suggests that, a combination of intervention strategies might be most effective in improving overall dietary intake. This is in line with the results of our study.

In our study we observed some unexpected results. First, we added the recommended daily amount of fruit and vegetables for all household members for 7 days in the FV^+^ condition and in the Snacks^–^ + FV^+^ condition. These amounts were however not fully reflected in mean daily intakes of fruit and vegetables of the food bank recipients who participated in our study in these conditions. Several explanations for this discrepancy exist. We cannot exclude that participants (1) hoarded the non-perishable supplied vegetables, (2) underreported their fruit and vegetable intake, or (3) gave away fruit and vegetables to, or exchanged fruit and vegetables for other food products with participants in other conditions or non-participating food bank recipients. Also, from literature it is known that adult caregivers may sacrifice their own diet to avoid that their children should experience hunger [29], and women may be the first to compromise their diet in an unhealthy way, to protect their children and partner when the family faces threats to their food supply [30, 31]. Although fruit and vegetable intakes were lower than theoretically could be expected in the FV^+^ condition and in the Snacks^–^ + FV^+^, still a significant increase in fruit and vegetable consumption was reported, which is known to be associated with recognizable decreases in morbidity and mortality [32, 33]. Second, we found unexpected results with regard to the mean daily food group intakes of pulses and sugar, candy, sweet filling and sweet sauces in the Snacks^–^ condition. In this intervention condition, unhealthy snacks (e.g., potato chips, cookies, chocolate) were replaced by staple foods such as pasta and rice. Therefore, we did not expect a statistically significant higher intake of sugar, candy, sweet filling and sweet sauces than in the Snacks^–^ + FV^+^ condition, nor a statistically significant higher intake of pulses in this condition than in both the control condition and the Snacks^–^ + FV^+^ condition. Perhaps, purchase and storage behavior of the participants could explain these results, but unfortunately these were not measured.

Strengths of this study include the comparison of several intervention conditions developed with input from food bank recipients, pilot-testing of the intervention conditions, the use of a randomized controlled trial with intention-to-treat analysis and a very low attrition rate (5.6%). Furthermore, by adding fruit, vegetables and staple foods to and removing unhealthy snacks (i.e., unhealthy foods) from the standard food parcel, we made it relatively easy for participants to improve dietary intake.

Limitations of this study should also be noted. The researchers as well as the participants were not blinded regarding allocation to the research conditions. Still, participants were not aware of the exact research aim. Furthermore, participants within one food bank were allocated to all possible intervention sequences. Therefore, participants could exchange or give away foods typical for their intervention condition, which could have led to an underestimation of the intervention effect. When using cluster randomizing, this would not have been the case. Also, we supplied some non-perishable products, such as non-perishable vegetables (for 3 days maximum), pasta and rice. We cannot exclude that participants hoarded these products which may have led to an underestimation of the vegetables, pasta and rice intake. However, to avoid hoarding, final measurements took place 2 weeks before the end of the intervention. Furthermore, we strived to collect dietary intake data of three days from each participant, for both intervention periods. Since we experienced that this was not always feasible in the first intervention period we decided to strive to collect dietary intake data of two days from each participant in the second intervention period. However, we used (several) multiple pass 24-h dietary recalls to collect dietary intake data. Previous studies showed that this method is considered most accurate to assess the overall energy, carbohydrate, protein, fat, or nutrient intake in low-income households [20, 34, 35], though underreporting is often observed in all self-reporting of dietary intake [36, 37]. In addition, we removed unhealthy snacks from the food parcels (e.g. chocolate, cookies, potato chips), but intake of snacks was measured through two food groups (i.e. salty and sweet) as defined in the NEVO-table [24]. These food groups are a bit broader and therefore also include healthy snacks such as nuts, and sweet filling such as jam. This may have led to an overestimation of unhealthy snack intake in our study. A final limitation of our study is that participants were self-selected. Therefore, we do not know whether the participants in our study were representative for the average food bank recipient and consequently bias may have occurred.

This study shows that improving the dietary quality of the food parcels positively impacts the dietary intake of Dutch food bank recipients. The results of this study revealed that adding fruit and vegetables to the standard food parcels, either in combination with replacing unhealthy snacks by staple foods or not, was an effective intervention strategy to increase daily fruit, vegetables and fiber intakes, and to decrease total fat intake. The intervention strategy in which unhealthy snacks from the food parcel were replaced by staple foods did not seem to be effective in improving dietary intake, it even seems to worsen the quality of food bank recipient's diet. Longer term studies are needed to assure the sustainability of the changes. With this knowledge we can further develop effective strategies that can be easily applied by food banks to improve dietary intake of Dutch food bank recipients.

## Abbreviations

SES: Socioeconomic status
24-h: 24-Hour
BMI: Body mass index
MPM: Multiple-pass method
SD: Standard deviation
95%CI: 95% Confidence interval
SNAP: Supplemental nutrition assistance program

## References

[1] Andreyeva T, Tripp AS, Schwartz MB (2015). Dietary quality of Americans by supplemental nutrition assistance program participation status: a systematic review. Am J Prev Med.

[2] Leung CW, Ding EL, Catalano PJ, Villamor E, Rimm EB, Willett WC (2012). Dietary intake and dietary quality of low-income adults in the supplemental nutrition assistance program. Am J Clin Nutr.

[3] Simmet A, Depa J, Tinnemann P, Stroebele-Benschop N (2017). The dietary quality of food pantry users: a systematic review of existing literature. J Acad Nutr Diet.

[4] Robaina KA, Martin KS (2013). Food insecurity, poor diet quality, and obesity among food pantry participants in Hartford, CT. J Nutr Educ Behav.

[5] Castetbon K, Mejean C, Deschamps V, Bellin-Lestienne C, Oleko A, Darmon N, Hercberg S (2011). Dietary behaviour and nutritional status in underprivileged people using food aid (ABENA study, 2004–2005). J Hum Nutr Diet.

[6] Depa J, Gyngell F, Muller A, Eleraky L, Hilzendegen C, Stroebele-Benschop N (2018). Prevalence of food insecurity among food bank users in Germany and its association with population characteristics. Prev Med Rep.

[7] Dave JM, Thompson DI, Svendsen-Sanchez A, Cullen KW (2017). Perspectives on barriers to eating healthy among food pantry clients. Health Equity.

[8] Nagelhout GE, Hogeling L, Spruijt R, Postma N, de Vries H (2017). Barriers and facilitators for health behavior change among adults from multi-problem households: a qualitative study. Int J Environ Res Public Health.

[9] Neter J, Dijkstra S, Nicolaou M, Visser M, Brouwer I (2020) The role of food parcel use on dietary intake: perception of Dutch food bank recipients–a focus group study. Accepted for publication in Eur J Nutr 10.1017/S1368980019003823 PMC10200378 32066521

[10] Verpy H, Smith C, Reicks M (2003). Attitudes and behaviors of food donors and perceived needs and wants of food shelf clients. J Nutr Educ Behav.

[11] Efrati Philip D, Baransi G, Shahar DR, Troen AM (2018). Food-aid quality correlates positively with diet quality of food pantry users in the leket israel food bank collaborative. Front Nutr.

[12] Simmet A, Depa J, Tinnemann P, Stroebele-Benschop N (2017). The nutritional quality of food provided from food pantries: a systematic review of existing literature. J Acad Nutr Diet.

[13] Neter JE, Dijkstra SC, Visser M, Brouwer IA (2016). Dutch food bank parcels do not meet nutritional guidelines for a healthy diet. Br J Nutr.

[14] Rambeloson ZJ, Darmon N, Ferguson EL (2008). Linear programming can help identify practical solutions to improve the nutritional quality of food aid. Public Health Nutr.

[15] Neter JE, Dijkstra SC, Visser M, Brouwer IA (2014). Food insecurity among Dutch food bank recipients: a cross-sectional study. BMJ Open.

[16] Neter JE, Dijkstra SC, Dekkers ALM, Ocke MC, Visser M, Brouwer IA (2018). Dutch food bank recipients have poorer dietary intakes than the general and low-socioeconomic status Dutch adult population. Eur J Nutr.

[17] Gezondheidsraad (2006) Richtlijnen goede voeding 2006 - achtergronddocument. Publicatie nr A06/08. Den Haag: Gezondheidsraad

[18] Frank E, Dunlop AL (2000). What does a patient's outfit weight?. Fam Med.

[19] World Health Organisation (WHO) Expert Committee (1995). Physical status: the use and interpretation of anthropometry.

[20] Conway JM, Ingwersen LA, Moshfegh AJ (2004). Accuracy of dietary recall using the USDA five-step multiple-pass method in men: an observational validation study. J Am Diet Assoc.

[21] Moshfegh AJ, Rhodes DG, Baer DJ, Murayi T, Clemens JC, Rumpler WV, Paul DR, Sebastian RS, Kuczynski KJ, Ingwersen LA, Staples RC, Cleveland LE (2008). The US Department of Agriculture Automated Multiple-Pass Method reduces bias in the collection of energy intakes. Am J Clin Nutr.

[22] Raper N, Perloff B, Ingwersen L, Steinfeldt L, Anand J (2004). An overview of USDA’s Dietary Intake Data System. J Food Compos Anal.

[23] Donders-Engelen M, Heijden van der L, Hulshof K (2003) Maten, Gewichten en Codenummers 2003. Wageningen: Gezamenlijke produktie van Afdeling Humane Voeding, Wageningen Universiteit en TNO Voeding te Zeist

[24] RIVM (2010) NEVO-tabel. Nederlands Voedingsstoffen bestand 2010. Den Haag: RIVM

[25] Rossum van C, Fransen H, Verkaik-Kloosterman J, Buurma-Rethams E, Ocké M (2011) Dutch National Food Consumption Survey 2007–2010. Diet of children and adults aged 7 to 69 years. Bilthoven: National Institute for Public Health and the Environment

[26] Depa J, Wolf A, Rössler V, Weiffenbach J, Hilzendegen C, Stroebele-Benschop N (2019). The impact of providing fruits and vegetables to socially disadvantaged men. J Hunger Environ Nutr.

[27] Harnack L, Oakes JM, Elbel B, Beatty T, Rydell S, French S (2016). Effects of subsidies and prohibitions on nutrition in a food benefit program: a randomized clinical trial. JAMA Intern Med.

[28] Olsho LE, Klerman JA, Wilde PE, Bartlett S (2016). Financial incentives increase fruit and vegetable intake among supplemental nutrition assistance program participants: a randomized controlled trial of the USDA Healthy Incentives Pilot. Am J Clin Nutr.

[29] Radimer KL, Olson CM, Greene JC, Campbell CC, Habicht JP (1992). Understanding hunger and developing indicators to assess it in women and children. J Nutr Educ.

[30] Martin MA, Lippert AM (2012). Feeding her children, but risking her health: the intersection of gender, household food insecurity and obesity. Soc Sci Med.

[31] McIntyre L, Glanville NT, Raine KD, Dayle JB, Anderson B, Battaglia N (2003). Do low-income lone mothers compromise their nutrition to feed their children?. CMAJ.

[32] Bazzano LA, He J, Ogden LG, Loria CM, Vupputuri S, Myers L, Whelton PK (2002). Fruit and vegetable intake and risk of cardiovascular disease in US adults: the first national health and nutrition examination survey epidemiologic follow-up study. Am J Clin Nutr.

[33] Bellavia A, Larsson SC, Bottai M, Wolk A, Orsini N (2013). Fruit and vegetable consumption and all-cause mortality: a dose-response analysis. Am J Clin Nutr.

[34] Holmes B, Dick K, Nelson M (2008). A comparison of four dietary assessment methods in materially deprived households in England. Public Health Nutr.

[35] Johnson RK, Driscoll P, Goran MI (1996). Comparison of multiple-pass 24-hour recall estimates of energy intake with total energy expenditure determined by the doubly labeled water method in young children. J Am Diet Assoc.

[36] Freedman LS, Commins JM, Moler JE, Arab L, Baer DJ, Kipnis V, Midthune D, Moshfegh AJ, Neuhouser ML, Prentice RL, Schatzkin A, Spiegelman D, Subar AF, Tinker LF, Willett W (2014). Pooled results from 5 validation studies of dietary self-report instruments using recovery biomarkers for energy and protein intake. Am J Epidemiol.

[37] Lissner L, Troiano RP, Midthune D, Heitmann BL, Kipnis V, Subar AF, Potischman N (2007). OPEN about obesity: recovery biomarkers, dietary reporting errors and BMI. Int J Obes.

